# Early mobilisation versus delayed protocols after reverse total shoulder arthroplasty for nonfracture indications: A systematic review and meta‐analysis

**DOI:** 10.1002/jeo2.70449

**Published:** 2025-10-08

**Authors:** Napatpong Thamrongskulsiri, Thun Itthipanichpong, Tanawan Kongmalai, Pinkawas Kongmalai

**Affiliations:** ^1^ Sports Medicine Research Group, Faculty of Medicine Chulalongkorn University Bangkok Thailand; ^2^ Department of Orthopaedics, Faculty of Medicine Chulalongkorn University Bangkok Thailand; ^3^ Department of Anatomy, Faculty of Medicine Chulalongkorn University Bangkok Thailand; ^4^ Department of Medicine, Faculty of Medicine, Division of Endocrinology and Metabolism, Siriraj Hospital Mahidol University Bangkok Thailand; ^5^ Department of Orthopedics, Faculty of Medicine Kasetsart University Bangkok Thailand

**Keywords:** delayed mobilisation, early mobilisation, rehabilitation, reverse total shoulder arthroplasty, systematic review

## Abstract

**Purpose:**

This study aimed to compare clinical outcomes, range of motion, pain scores and complication rates between early and delayed mobilisation following reverse total shoulder arthroplasty (RTSA).

**Methods:**

A systematic review and meta‐analysis were conducted according to 2020 Preferred Reporting Items for Systematic Reviews and Meta‐Analyses guidelines. PubMed, Ovid Medline and Scopus databases were searched from inception through May 2025. Comparative studies evaluating early versus delayed mobilisation after RTSA were included. Methodological quality was assessed using the modified Coleman Methodology Score, while risk of bias was evaluated using the Newcastle‐Ottawa Scale for cohort studies and the Risk of Bias 2 tool for randomised controlled trials. Pooled outcomes included patient‐reported outcome scores, range of motion and postoperative complications.

**Results:**

Six studies with a total of 1763 patients were included. All included studies in this review investigated RTSA for nonfracture indications. Methodological quality ranged from fair to excellent across included studies. Meta‐analysis showed that early mobilisation was associated with statistically significantly greater improvements in forward flexion (mean difference [MD] of 4.36°), abduction (MD of 4.95°) and pain visual analogue scale scores (MD of –0.40) compared to delayed mobilisation. No statistically significant differences were found between groups in Constant scores, American Shoulder and Elbow Surgeons scores, external rotation, dislocation rates, or revision surgery. Notably, early mobilisation was associated with a lower incidence of postoperative fractures.

**Conclusion:**

Early mobilisation after RTSA may yield modest improvements in pain and shoulder motion, though below established MCID thresholds. Importantly, it was associated with a lower risk of postoperative fractures and did not increase other complications. These findings support the safety of early rehabilitation, while highlighting the limited clinical magnitude of benefit.

**Level of Evidence:**

Level III.

**Abbreviations**
AQOL‐4D: assessment of quality‐of‐life instrument
ASES: American Shoulder and Elbow Surgeons
CI: confidence interval
DLA: daily living activities
GSF: global shoulder function
MCID: minimal clinically important difference
MCMS: modified Coleman Methodology Score
MIRCT: massive irreparable rotator cuff tear
NOS: Newcastle‐Ottawa Scale
NR: not reported
OA: osteoarthritis
ON: osteonecrosis
OR: odds ratio
PRISMA: preferred reporting items for systematic reviews and meta‐analyses
PROSPERO: International Prospective Register of Systematic Reviews
RA: rheumatoid arthritis
RCTA: rotator cuff tear arthropathy
RoB 2: risk of bias 2
ROM: range of motion
RTSA: reverse total shoulder arthroplasty
SANE: single assessment numeric evaluation
SAS: shoulder activity scale
SSV: subjective shoulder value
VAS: visual analogue scale

## INTRODUCTION

Reverse total shoulder arthroplasty (RTSA) has become an increasingly popular surgical treatment for patients with various shoulder conditions, including cuff‐deficient shoulders, osteoarthritis, rheumatoid arthritis and fractures, offering substantial pain relief and functional improvement [3, 8]. Unlike anatomical total shoulder arthroplasty, the reverse prosthesis functions differently from a biomechanical standpoint, with its design medializing the centre of rotation, thereby shifting the primary responsibility for shoulder elevation from the rotator cuff to the deltoid muscle [5, 10, 19]. As such, while the success of RTSA is less dependent on rotator cuff integrity than anatomic total shoulder arthroplasty, preservation and healing of the remaining cuff—particularly the external rotators (infraspinatus and teres minor), and to a lesser extent the subscapularis—can further enhance prosthesis function and improve patient‐reported outcomes [2]. Because RTSA is a semiconstrained prosthesis, it offers intrinsic stability through its design, thereby further reducing reliance on the rotator cuff for joint stability. This biomechanical construct raises the question of whether prolonged postoperative immobilisation, as traditionally used to protect rotator cuff repairs, is necessary following RTSA.

Despite growing clinical experience with RTSA, there remains significant variability in postoperative rehabilitation protocols. Traditionally, patients were immobilised for 4–6 weeks postoperatively to protect soft tissue healing and avoid complications such as dislocation [13, 15]. However, advances in surgical techniques—particularly the omission of subscapularis repair—and improved implant designs have prompted some clinicians to question the need for prolonged immobilisation [16]. Recent studies suggest that earlier initiation of motion, or even direct active rehabilitation, may be safe and effective. Kornuijt et al. reported favourable outcomes with immediate active rehabilitation [16], Torrens et al. found no differences between immediate and 3‐week immobilisation [24], and Lee et al. demonstrated comparable or fewer complications with accelerated protocols [17]. Collectively, these findings indicate that early mobilisation may be both safe and beneficial, though evidence remains heterogeneous in quality.

The aim of this systematic review and meta‐analysis was to compare the clinical outcomes of early versus delayed mobilisation following RTSA for nonfracture indications, focusing on functional recovery, pain, complication rates and patient‐reported outcome measures. The authors hypothesised that early mobilisation after RTSA would not be associated with increased complications and may result in improved or comparable functional outcomes compared to delayed mobilisation protocols involving postoperative immobilisation.

## METHODS

### Search strategy

The author (N.T.), carried out comprehensive literature searches in PubMed, Ovid Medline and Scopus to identify studies comparing clinical outcomes of early versus delayed mobilisation following RTSA. To ensure both accuracy and completeness, each author performed the search separately, capturing all relevant studies published from the inception of each database through 23 May 2025. This systematic review was conducted in accordance with the 2020 preferred reporting items for systematic reviews and meta‐analyses (PRISMA) guidelines [20] and was prospectively registered with PROSPERO (ID: CRD420251054106). The search combined the terms ‘reverse total shoulder arthroplasty’ OR ‘reverse shoulder replacement’ OR ‘RTSA’ with ‘early mobilisation’ OR ‘delayed mobilisation’ OR ‘rehabilitation’ OR ‘immobilisation’. References of included studies were also screened, but no additional hand searches or expert contacts were performed. Complete database‐specific search strategies are provided in Supporting Information S1: Appendix A.

### Inclusion and exclusion criteria

Studies were included in this review if they met the following criteria: (1) clinical comparative research with a level of evidence between 1 and 3, (2) published in English, (3) directly assessed early versus delayed mobilisation following RTSA (with ‘early’ generally defined as immediate initiation of motion to within 2–3 weeks postoperatively, and ‘delayed’ as initiation at approximately 3–6 weeks postoperatively), (4) reported clinical outcomes or treatment‐related complications and (5) provided full‐text availability. Studies were excluded if they focused on basic science or biomechanics, were case series or individual case reports, or were review articles.

### Data extraction

Two authors (N.T. and P.K.) independently screened the titles, abstracts, and full texts to determine study eligibility using Covidence (https://www.covidence.org/). Any disagreements were resolved through consultation with a third author (T.I.). From each included study, the following data were extracted: (1) publication details, (2) level of evidence, (3) patient demographics, (4) outcome measures, (5) surgical technique, (6) postoperative rehabilitation protocol and (7) reported clinical outcomes.

### Methodological quality and risk of bias assessment

Methodological quality was appraised using the modified Coleman Methodology Score (MCMS), a validated instrument that evaluates the methodological soundness of clinical research [6]. This scoring system considers elements such as study design, patient selection criteria, duration and adequacy of follow‐up, and the robustness of statistical analysis. Scores range from 0 to 100, with values under 50 classified as poor, 50–69 as fair, 70–84 as good and 85 or above as excellent.

Following the methodological quality assessment, the risk of bias was evaluated using tools appropriate for each study design. For cohort studies, the Newcastle‐Ottawa Scale (NOS) was used [22], which assesses studies across three main domains: selection (maximum four points), comparability (maximum two points) and outcome (maximum three points), for a total score of up to nine points. Studies awarded 7–9 points were considered to have a low risk of bias, those with 4–6 points as moderate risk, and those with fewer than 4 points as high risk of bias. For randomised controlled trials, the Risk of Bias 2 (RoB2) tool was employed [23]. This tool evaluates five domains: (1) bias arising from the randomisation process, (2) bias due to deviations from intended interventions, (3) bias due to missing outcome data, (4) bias in measurement of the outcome and (5) bias in selection of the reported result. Each domain is rated as having low risk of bias, some concerns, or high risk of bias. An overall risk of bias judgement is then derived for each study based on the ratings across all domains. Two reviewers (N.T. and T.K.) independently rated each study, and any disagreements were resolved with the involvement of a third reviewer (T.I.).

### Statistical analysis

The meta‐analysis was analysed using RevMan for Windows (Cochrane, version 5.4.1). Odds ratios (ORs) with 95% confidence intervals (CIs) were calculated for dichotomous outcomes, while mean differences (MDs) with their corresponding 95% CIs were calculated for continuous outcomes. Statistical heterogeneity was assessed using the chi‐square test, with a *I* ^2^ of more than 50% considered indicative of significant heterogeneity among the studies. A random‐effects model was applied when statistical evidence of heterogeneity was present. Conversely, a fixed‐effects model was used when no such heterogeneity was detected.

## RESULTS

### Study selection

**Figure 1:**
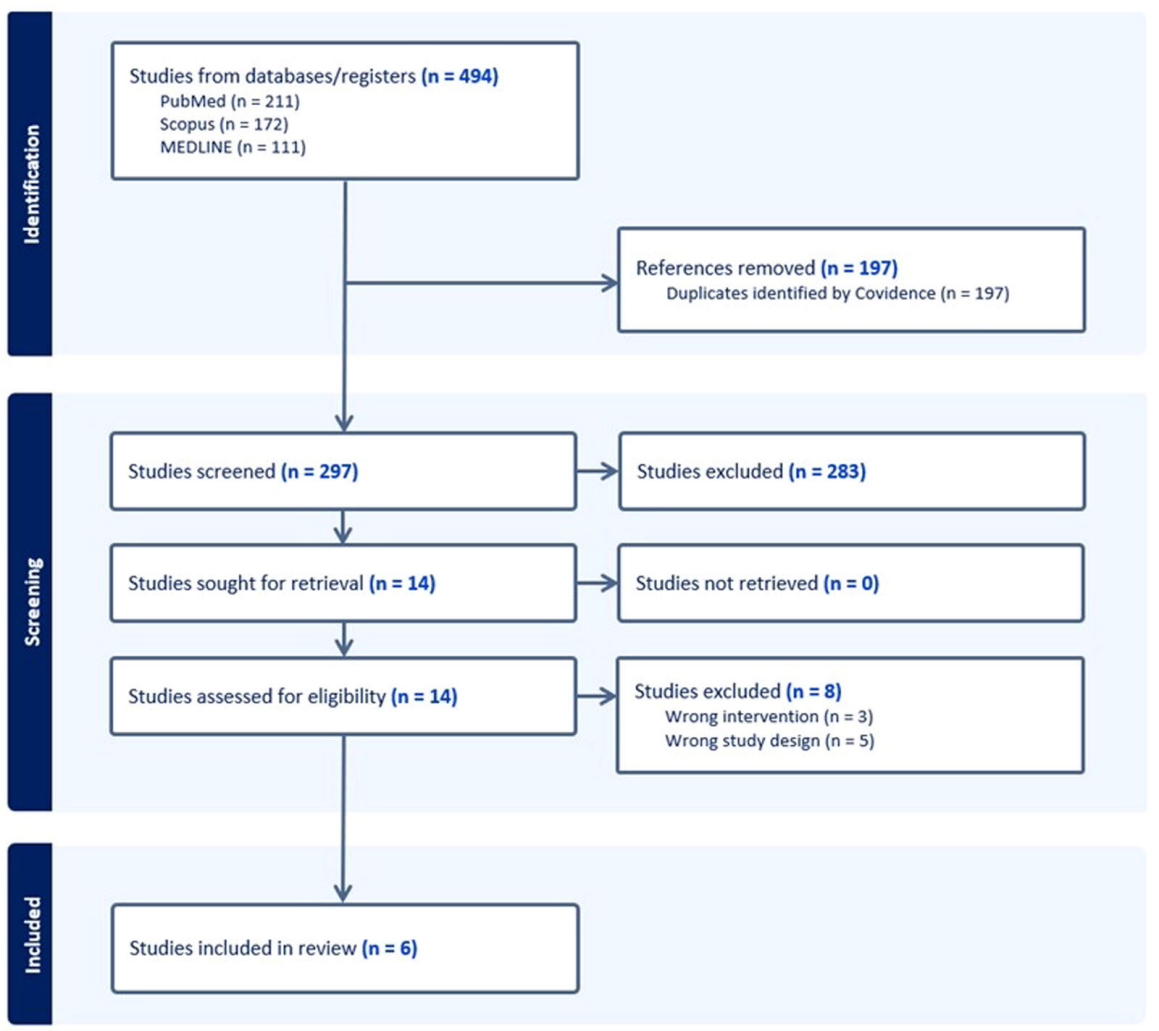
A preferred reporting items for systematic reviews and meta‐analyses flow diagram showing the search and selection process.

The initial search yielded 494 records. After removing 197 duplicates with Covidence (https://www.covidence.org), 297 records remained for title and abstract screening. Of these, 283 were excluded. Fourteen full‐text articles were assessed for eligibility, and eight were excluded. Finally, six studies [1, 7, 11, 12, 17, 24] met the inclusion criteria and were included in this review (Figure 1).

The systematic review included six studies [1, 7, 11, 12, 17, 24] comprising a total of 1763 patients who underwent RTSA, comparing early versus delayed rehabilitation protocols. The studies varied in level of evidence (1–3) and follow‐up duration (1–2 years), with mean patient ages ranging from approximately 59.7–75.5 years and body mass index from 27.4 to 32.6 kg/m² (Table 1). Diagnoses across studies included rotator cuff tear arthropathy, osteoarthritis, rheumatoid arthritis, massive irreparable rotator cuff tear and postinfectious (Table 2). Surgical techniques varied slightly across studies, with most employing a deltopectoral approach and lateralized implant designs, and some involving subscapularis tendon repair. Rehabilitation protocols in the early group generally initiated passive and/or active‐assisted motion within the first few weeks postoperatively, whereas the delayed group typically underwent longer immobilisation periods ranging from 3 to 6 weeks before initiating movement (Table 3).

**Table 1:** Characteristics of included studies: Level of evidence, mean follow‐up, mean age, and body mass index.

Lead author	LOE	Sample size		Mean FU, years		Mean age, years		BMI, kg/m^2^	
		Early	Delayed	Early	Delayed	Early	Delayed	Early	Delayed
Hagen (2020)	I	30	35	2	2	68.3 ± 10.5	69.4 ± 7.5	30.84	28.87
Edward (2021)	I	30	33	1	1	75.1 ± 6.1	73.6 ± 6.1	32.6	28.8
Lee (2021)	III	Immobilisation ‐ no: 118 − 3 weeks: 125	114	1	1	Immobilisation ‐ no: 76 − 3 weeks: 74	76	NR	NR
Alben (2023)	III	684	276	1.6 ± 1.8	1.7 ± 1.8	71.1 ± 9.3	70.5 ± 8.4	29.80	30.50
Hochberger (2024)	II	25	35	1	1	59.7 ± 7.0	75.5 ± 5.7	28.50	27.40
Torrens (2025)	I	26	27	2	2	72.1 ± 7.8	74.8 ± 7.2	NR	NR

Abbreviations: BMI, body mass index; FU, follow‐up; LOE, level of evidence; NR, not reported.

**Table 2:** Details of included studies: Diagnosis, outcome measures, and MCMS.

Lead author	Diagnosis		Outcome measures	MCMS
	Early	Delayed		
Hagen (2020)	RCTA, revision	RCTA, revision, fracture malunion	ASES, ROM, complication, revision, narcotic use, radiographic results (osteolysis, scapular notching)	88
Edward (2021)	NR	NR	ASES, pain VAS, GSF, SANE, AQOL‐4D, SAS, ROM, complication	88
Lee (2021)	RCTA, OA, RA, revision, MIRCT, instability, arthropathy, postinfection	RCTA, OA, RA, revision, MIRCT	Constant, SSV, SANE, satisfaction, ROM, complications, revision, radiographic results (osteolysis, scapular notching)	73
Alben (2023)	RCTA, OA, instability	RCTA, OA, instability	ROM, pain VAS, complication, revision, radiographic results (osteolysis, scapular notching)	64
Hochberger (2024)	RCTA, OA, pathologic fracture, infection	RCTA, OA, pathologic fracture, instability, ON	ASES, SST, pain VAS, ROM	63
Torrens (2025)	RCTA	RCTA	Constant, pain VAS, DLA, ROM, complication	85

Abbreviations: AQOL‐4D, assessment of quality‐of‐life instrument; ASES, American Shoulder and Elbow Surgeons; DLA, daily living activities; GSF, global shoulder function; MCMS, modified Coleman Methodology Score; MIRCT, massive irreparable rotator cuff tear; NR, not reported; OA, osteoarthritis; ON, osteonecrosis; RA, rheumatoid arthritis; RCTA, rotator cuff tear arthropathy; ROM, range of motion; SANE, single assessment numeric evaluation; SAS, shoulder activity scale; SWB, subjective well‐being; VAS, visual analogue scale.

**Table 3:** Details of included studies: Inclusions, exclusions, surgical technique, and postoperative rehabilitation.

Lead author	Inclusions	Exclusions	Surgical technique	Postoperative rehabilitation	
				Early	Delayed
Hagen (2020)	– Patient who underwent RTSA	NR	– Approach: deltopectoral – Subscapularis: no repair – Implant: Zimmer Biomet Trabecular Metal reverse shoulder system (Zimmer Biomet, Warsaw) – Implant design: medialized design	– Immediate physical therapy for passive and active ROM – Weaning of sling use as tolerated but no resistance training for 6 weeks	– 6 weeks sling immobilisation – No passive or active motion of the shoulder for 6 weeks
Edward (2021)	– Aged 55–85 years – Symptomatic MIRCT or OA	– Fractures or dislocations – Revision – Pre‐existing conditions associated with upper extremity pain – Infection – Peripheral nerve compression syndrome – Cervical neuropathy – Inflammatory arthritis	– Approach: deltopectoral – Subscapularis: no repair – Implant: Equinoxe Reverse Shoulder Design (Exactech, Gainesville) – Implant design: lateralized design	– Weeks 0–2: Sling with education – Weeks 2–6: Passive and selected active‐assisted ROM; isometric deltoid and external rotator strengthening – Weeks 6–12: Active‐assisted and active ROM; early external rotator strengthening in ≥45° abduction – Weeks 12–20: Weekly supervised sessions + daily home program; focused on deltoid, rotators, and scapulothoracic muscles using low‐load, high‐rep protocol for endurance	– Weeks 0–2: Sling only, no exercises – Weeks 2–6: Passive ROM – Weeks 6–12: Active‐assisted ROM – Post‐12 weeks: No further formal rehab; encouraged to continue independently
Lee (2021)	– RCTA – OA – RA – Failed rotator cuff repair – MIRCT – Fracture sequela – Instability arthropathy	– Acute proximal humerus fracture – Intraoperative complication–fracture	– Approach: anterosuperior – Subscapularis: repair – Implant: Stemless metaphyseal RTSA prosthesis (Verso, Innovative Design Orthopaedics) – Implant design: lateralized design	– 3 weeks sling immobilisation (*n* = 125) – No immobilisation (*n* = 118)	6 weeks sling immobilisation
Alben (2023)	– Patient who underwent RTSA – Minimum 1 year follow‐up	– Revision – Proximal humerus fracture	– Approach: deltopectoral – Subscapularis: Early: Repair (*n* = 266); no repair (*n* = 4); NR (*n* = 6), delayed: repair (*n* = 473); no repair (*n* = 54); NR (*n* = 157) – Implant: NR – Implant design: lateralized design: lateralized design, 78.3%; medialized design, 21.7%	2 weeks sling immobilisation	6 weeks sling immobilisation
Hochberger (2024)	– Patient who underwent RTSA – Aged >18 years	– Prior arthroplasty procedure to the affected shoulder – Revision – Neuromuscular diseases	– Approach: deltopectoral – Subscapularis: repair – Implant: Tornier Humeral Perform or Ascend Flex (humeral side) and Aequalis Revers 2 or Perform Revers (glenoid side) (Stryker, Bloomington) – Implant design: lateralized design	4 weeks sling immobilisation	6 weeks sling immobilisation
Torrens (2025)	– Aged 18–85 years – Patient who underwent RTSA	– Previous shoulder surgeries on the affected side – Significant glenoid defects – Cognitive deterioration – Impossible to conduct a proper rehabilitation program – Unwillingness to participate	– Approach: deltopectoral – Subscapularis: repair – Implant: Humelock Reversed (Fx Shoulder Solutions) – Implant design: lateralized design	No immobilisation	3 weeks sling immobilisation

Abbreviations: MIRCT, massive irreparable rotator cuff tear; NR, not reported; OA, osteoarthritis; RA, rheumatoid arthritis; RCTA, rotator cuff tear arthropathy; ROM, range of motion; RTSA, reverse total shoulder arthroplasty.

### Methodological quality and risk of bias

The methodological quality of the included studies was assessed using the MCMS. The scores ranged from 63 to 88, reflecting a methodological quality that varied from fair to excellent across the studies (Table 2).

The risk of bias in the cohort studies was assessed using the NOS. All three studies [1, 12, 17] were rated as having a low risk of bias. While Lee et al. [17] and Alben et al. [1] each scored 7 out of 9 due to limitations in selection of the nonexposed cohort and adequacy of follow‐up, Hochberger et al. [12] achieved the maximum score of 9, indicating high methodological quality across all NOS domains (Table 4).

**Table 4:** Results of the Newcastle‐Ottawa Scale for assessing the risk of bias in cohort studies.

Lead author	Selection				Comparability		Outcome			Risk of bias
	REC	SNEC	AE	DO	BF	OF	AO	FU	AFU	
Lee (2021)	1	0	1	1	1	1	1	1	0	Low
Alben (2023)	1	0	1	1	1	1	1	1	0	Low
Hochberger (2024)	1	1	1	1	1	1	1	1	1	Low

Abbreviations: AE, ascertainment of exposure; AFU, adequacy of follow‐up of cohorts; AO, assessment of outcome; BF, study controls for age; DO, demonstration that outcome of interest was not present at the start of the study; FU, follow‐up long enough for outcomes to occur; OF, study controls for any other factors (sex, number of comorbidities, BMI and fracture type); REC, representativeness of the exposed cohort; SNEC, selection of the nonexposed cohort.

**Figure 2:**
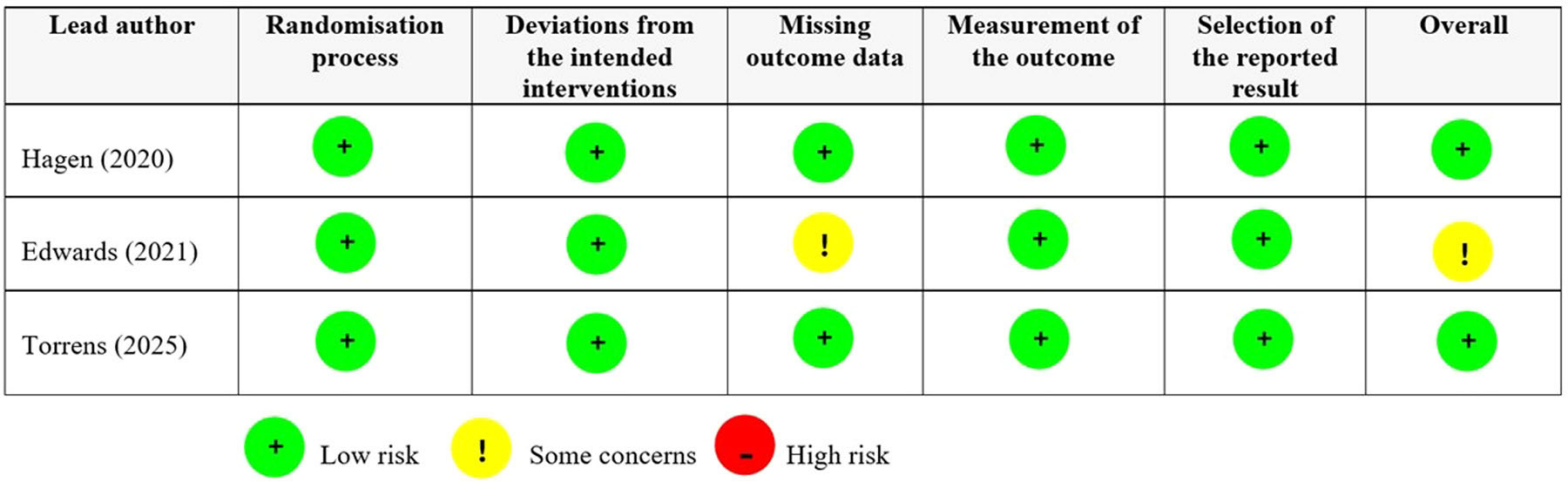
Results of the risk of bias 2 tool for assessing the risk of bias in randomised controlled trials.

The risk of bias in the included randomised controlled trials was assessed using the RoB 2 tool for randomised controlled trials. Of the three studies [7, 11, 24], two [11, 24] were judged to have a low overall risk of bias, while one study [7] was rated as having some concerns, primarily due to issues related to missing outcome data. All studies showed a low risk of bias in the domains of randomisation, deviations from intended interventions, measurement of the outcome and selection of the reported result (Figure 2).

### Patient‐reported outcome scores

**Figure 3:**
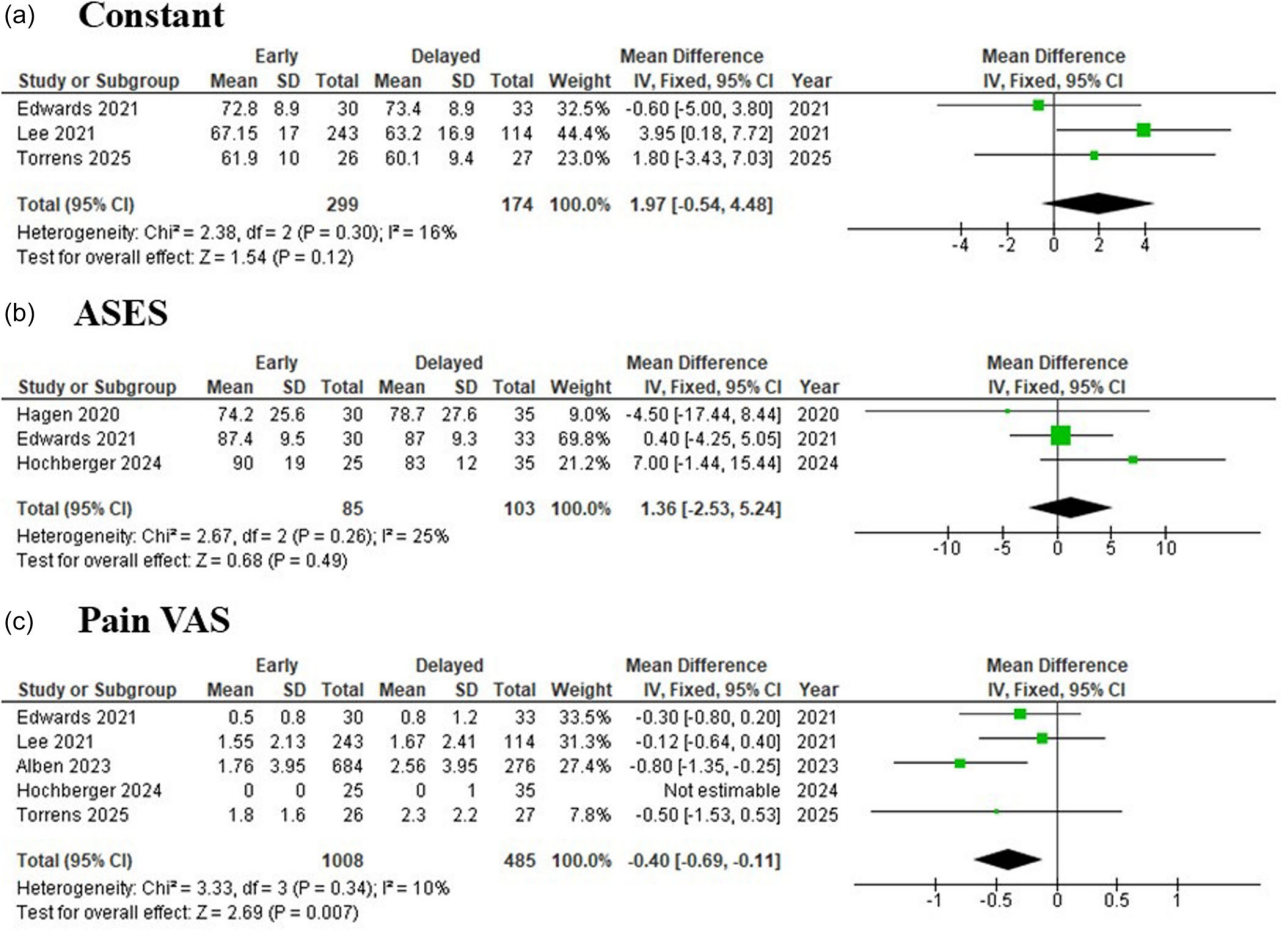
The forest plot summarises the findings comparing early versus delayed mobilisation following reverse total shoulder arthroplasty, focusing on three key outcome measures: (a) Constant score, (b) ASES score and (c) Pain VAS. ASES, American Shoulder and Elbow Surgeons; IV, inverse variance; VAS; visual analogue scale.

Three studies [7, 17, 24], including a total of 473 patients (299 in the early group and 174 in the delayed group), reported Constant scores. The pooled meta‐analysis demonstrated no statistically significant difference, with a MD of 1.97 (95% CI: −0.54 to 4.48; *p* = 0.12). Given the low heterogeneity (*I*² = 16%), a fixed‐effect model was applied (Figure 3a).

Three studies [7, 11, 12], including a total of 188 patients (85 in the early group and 103 in the delayed group), reported American Shoulder and Elbow Surgeons (ASES) scores. The pooled meta‐analysis demonstrated no statistically significant difference, with a MD of 1.36 (95% CI: −2.53 to 5.24; *p* = 0.49). Given the low heterogeneity (*I*² = 25%), a fixed‐effect model was applied (Figure 3b).

Four studies [1, 7, 17, 24], including a total of 1493 patients (1008 in the early group and 485 in the delayed group), reported pain visual analog scale (VAS). The pooled meta‐analysis demonstrated a statistically significant difference in favour of early mobilisation, with a MD of −0.40 (95% CI: −0.69 to −0.11; *p* = 0.007). Given the low heterogeneity (*I*² = 10%), a fixed‐effect model was applied (Figure 3c).

### Range of motion

**Figure 4:**
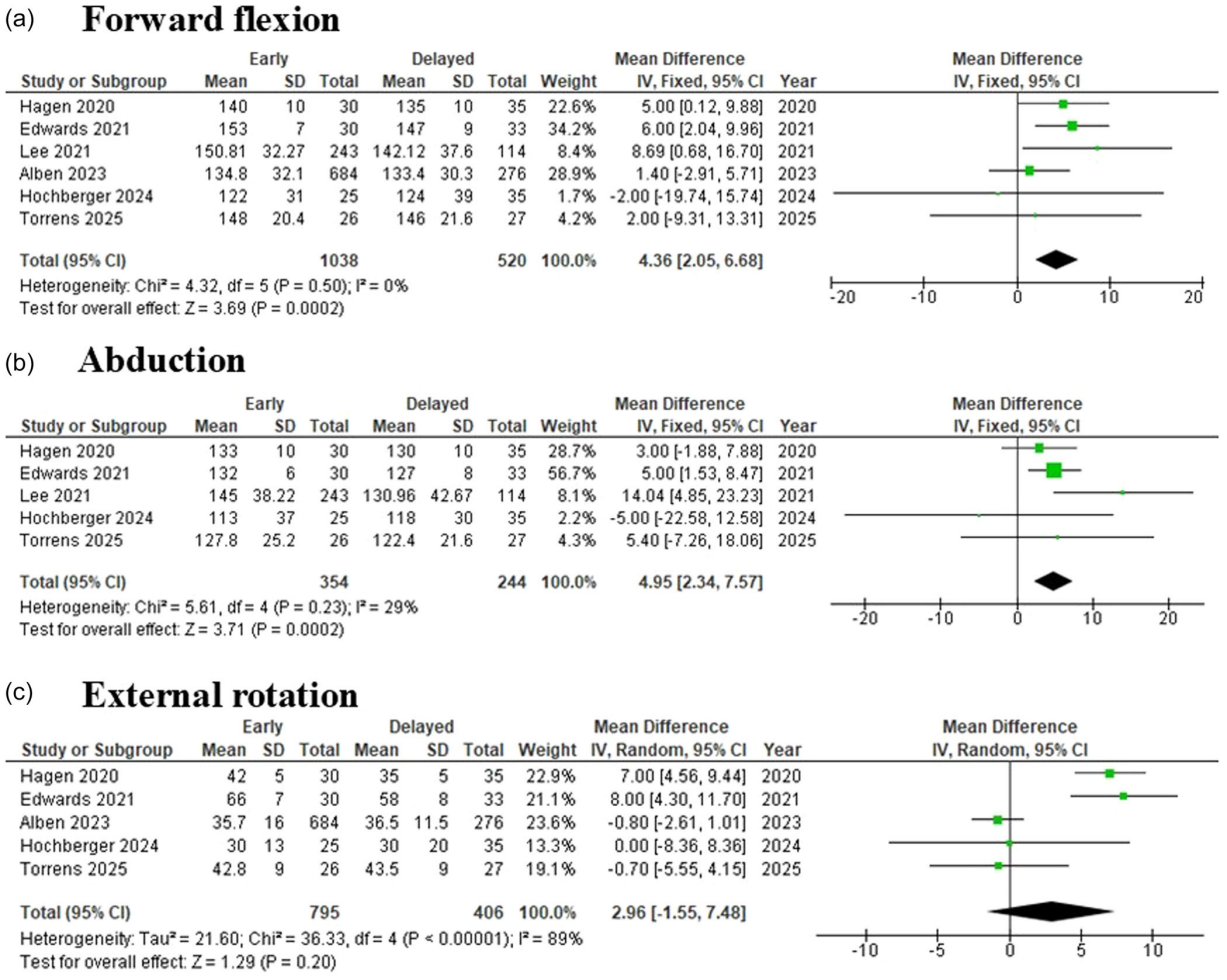
The forest plot summarises the findings comparing early versus delayed mobilisation following reverse total shoulder arthroplasty, focusing on three key outcome measures: (a) forward flexion, (b) abduction and (c) external rotation. IV, inverse variance.

Six studies [1, 7, 11, 12, 17, 24], including a total of 1558 patients (1038 in the early group and 520 in the delayed group), reported forward flexion. The pooled meta‐analysis demonstrated a statistically significant difference in favor of early mobilisation, with a MD of 4.36 (95% CI: 2.05–6.68; *p* = 0.0002). Heterogeneity was negligible (*I*² = 0%), so a fixed‐effect model was applied (Figure 4a).

Five studies [7, 11, 12, 17, 24], including 598 patients (354 in the early group and 244 in the delayed group), reported abduction range of motion. The pooled meta‐analysis showed a statistically significant difference in favor of early mobilisation, with a MD of 4.95 (95% CI: 2.34–7.57; *p* = 0.0002). Heterogeneity was low (*I*² = 29%), and a fixed‐effect model was used (Figure 4b).

Five studies [1, 7, 11, 12, 24], with a total of 1201 patients (795 in the early group and 406 in the delayed group), assessed external rotation. The pooled meta‐analysis demonstrated no statistically significant difference, with a MD of 2.96 (95% CI: −1.55 to 7.48; *p* = 0.20). Due to substantial heterogeneity (*I*² = 89%), a random‐effects model was applied (Figure 4c).

Although statistically significant improvements were observed in forward flexion (MD 4.36°) and abduction (MD 4.95°), these changes were smaller than the reported minimal clinically important difference (MCID) for shoulder range of motion (10°–15°) [21]. Similarly, the improvement in pain VAS scores (MD –0.40) did not exceed the MCID threshold for pain relief in shoulder conditions (1.4–2.0 points) [4].

### Complications

**Figure 5:**
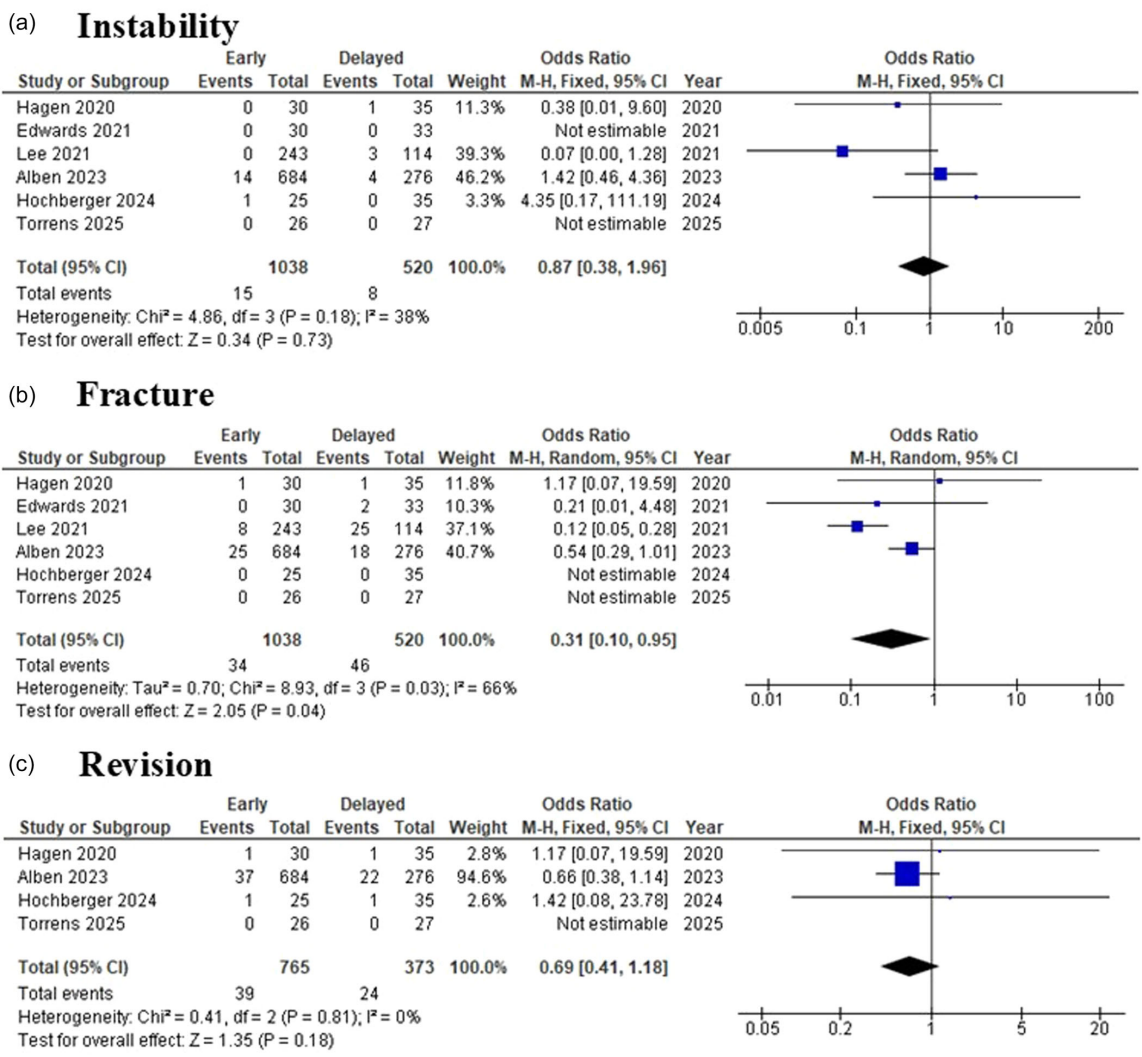
The forest plot summarises the findings comparing early versus delayed mobilisation following reverse total shoulder arthroplasty, focusing on three key outcome measures: (a) instability, (b) fracture and (c) revision. M–H, Mantel–Haenszel.

Six studies [1, 7, 11, 12, 17, 24], including a total of 1558 patients (1038 in the early mobilisation group and 520 in the delayed group), reported on postoperative instability, defined as prosthetic dislocation. The overall instability rates in the early and delayed groups were 1.4% and 1.5%, respectively. The pooled meta‐analysis demonstrated no statistically significant difference in dislocation rates between early and delayed mobilisation groups, with an OR of 0.87 (95% CI: 0.38 to 1.96; *p* = 0.73). Heterogeneity was low (*I*² = 38%), and a fixed‐effect model was used for analysis (Figure 5a).

Six studies [1, 7, 11, 12, 17, 24] evaluated postoperative fracture‐related complications, including acromial stress fractures, scapular spine stress fractures and periprosthetic fractures. A total of 1558 patients (1038 in the early mobilisation group and 520 in the delayed group) were included in this analysis. The overall fracture‐related complication rates in the early and delayed groups were 3.3% and 8.8%, respectively. The pooled results revealed a statistically significant lower risk of fracture in the early mobilisation group, with an OR of 0.31 (95% CI: 0.10–0.95; *p* = 0.04). Moderate heterogeneity was observed (*I*² = 66%), and a random‐effects model was applied (Figure 5b).

Four studies [1, 11, 12, 24] with a total of 1138 patients (765 early, 373 delayed) reported rates of revision surgery following RTSA. The overall revision rates in the early and delayed groups were 5.1% and 6.4%, respectively. The pooled meta‐analysis showed no statistically significant difference between groups, with an OR of 0.69 (95% CI: 0.41–1.18; *p* = 0.18). Heterogeneity was minimal (*I*² = 0%), justifying the use of a fixed‐effect model (Figure 5c).

## DISCUSSION

The most important finding of this study is that early rehabilitation after RTSA improves forward flexion and abduction, produces a small but statistically significant reduction in postoperative pain, and does so without increasing the risk of major complications. In this review, ‘early’ was generally defined as initiation of motion immediately or within 2–3 weeks postoperatively, whereas ‘delayed’ referred to initiation at approximately 3–6 weeks postoperatively. Our findings support previous reports that early motion after RTSA is safe. Forward flexion improved by 4.36° and abduction by 4.95° with early mobilisation, though both values were below the MCID for shoulder ROM (10°–15°) [21], suggesting limited clinical relevance. Most studies used lateralized implants without routine subscapularis repair [1, 7, 11, 12, 17, 24], reinforcing reduced need for prolonged immobilisation.

Although early mobilisation demonstrated statistically significant improvements in forward flexion (MD 4.36°) and abduction (MD 4.95°) compared with delayed mobilisation, these magnitudes of change are notably smaller than the MCID commonly reported for shoulder ROM, which ranges from approximately 10°–15° [21]. This suggests that while the differences are statistically significant, they may not translate into perceptible functional gains for most patients in daily activities. Similarly, the improvement in pain VAS scores (MD –0.40) falls below the MCID for pain relief in shoulder conditions, often estimated at around 1.4–2.0 points [4]. Thus, although measurable in a pooled analysis, these benefits are unlikely to be clinically meaningful.

Interestingly, when examining fractures specifically—including periprosthetic, acromial and scapular spine fractures—early rehabilitation was not only noninferior but also associated with a significantly lower risk. Meta‐analysis revealed a 69% reduction in fracture incidence among patients undergoing early rehabilitation (OR = 0.31, 95% CI 0.10–0.95, *p* = 0.04). Although this result warrants cautious interpretation due to substantial heterogeneity (*I*² = 66%), one possible explanation is improved proprioception and postural control. Lee et al. [17] proposed that early mobilisation may enhance neuromuscular coordination and balance, thereby reducing fall risk—a major contributor to periprosthetic fractures [13, 17]. Enhanced deltoid activation and shoulder girdle strength may also improve protective reflexes during a fall, potentially mitigating fracture severity. Additionally, gradual and guided loading of the upper extremity during early rehabilitation may promote bone quality through mechanotransduction, lowering susceptibility to stress fractures such as those of the acromion or scapular spine [9]. Complication risk after RTSA is closely related to implant design. In medialized implants, where subscapularis repair is often feasible, prolonged immobilisation may be warranted to protect the repair and maintain implant stability [2]. In contrast, lateralized implants provide greater intrinsic stability through their design, frequently precluding subscapularis repair, and may therefore allow for earlier mobilisation without increasing the risk of instability. These implant‐specific considerations should be integrated into postoperative rehabilitation planning to balance safety with functional recovery.

This review highlights that the structure and content of rehabilitation programs may be more influential than timing alone. Studies incorporating supervised protocols with targeted deltoid activation and progressive loading (e.g., Edwards et al. [7], Lee et al. [17], Hochberger et al. [12]) demonstrated superior functional outcomes compared to less structured approaches that emphasised early sling removal without progressive exercise (e.g., Alben et al. [1], Torrens et al. [24]). In older adults, where early return to independence is often a clinical priority, structured early rehabilitation may support faster recovery without increasing the risk of complications such as instability, revision, or fractures [14]. When early mobilisation is paired with exercises focused on deltoid activation and functional movement [18, 25], recovery may be further enhanced. These considerations are particularly relevant for patients receiving lateralized implant designs or those without subscapularis repair. Individualised rehabilitation protocols—tailored to patient goals, surgical technique and comorbidities—should therefore be emphasised in postoperative planning. For older adults, early mobilisation protocols can prioritise safe, supervised, deltoid‐focused strengthening, balance training and gradual functional task integration to accelerate independence while minimising fall risk. For patients without subscapularis repair, emphasis on controlled forward elevation and abduction in the early phases may optimise functional gains while avoiding undue stress on anterior soft tissues. Incorporating structured progression criteria—such as pain tolerance, movement quality and absence of instability—can help tailor rehabilitation to individual patient profiles. However, these findings should be interpreted with caution, as the improved functional outcomes cannot be attributed solely to the rehabilitation protocol and may also be influenced by implant design, surgical technique, and surgical indication.

This study has several strengths. It is the first to synthesise comparative studies comparing early and delayed rehabilitation after RTSA. The inclusion of a large patient cohort, validated outcome measures and analysis of both functional outcomes and complications enhances its clinical relevance. Moreover, the methodological quality of the included studies ranged from fair to excellent (MCMS), with most showing low risk of bias, supporting the overall credibility of the findings.

However, several limitations should be acknowledged. First, the definition of ‘early rehabilitation’ varied among studies, ranging from immediate motion to initiation within 2–3 weeks postoperatively. This heterogeneity limits the precision of subgroup comparisons and could mask true differences in outcomes. Second, while statistically significant, many of the observed improvements fell below established MCID—typically 10°–15° for shoulder motion and 1–2 points for pain VAS—raising questions about their clinical relevance [4, 21]. Third, most studies reported outcomes at short‐ to mid‐term follow‐up (1–2 years), with limited evidence on long‐term durability. Fourth, The substantial heterogeneity observed for external rotation outcomes (I² = 89%) may be attributable to variations in surgical techniques (e.g., differences in humeral version or tensioning of the deltoid and soft tissues), patient demographics, and differences in rehabilitation protocols. These factors likely contributed to the inconsistent outcome improvements reported across studies. Finally, early mobilisation was associated with a lower overall incidence of postoperative fractures compared with delayed mobilisation. When stratified by fracture type, acromial and scapular spine fractures were the most commonly reported, followed by periprosthetic fractures.

## CONCLUSION

Early mobilisation after RTSA may yield modest improvements in pain and shoulder motion, though below established MCID thresholds. Importantly, it was associated with a lower risk of postoperative fractures and did not increase other complications. These findings support the safety of early rehabilitation, while highlighting the limited clinical magnitude of benefit.

## AUTHOR CONTRIBUTIONS

Pinkawas Kongmalai contributed to the conception and design of the study. Literature search, study selection, and data extraction were independently performed by Napatpong Thamrongskulsiri and Pinkawas Kongmalai, with conflict resolution by Thun Itthipanichpong. Methodological quality assessment and risk of bias evaluation were conducted by Napatpong Thamrongskulsiri and Tanawan Kongmalai, with adjudication by Thun Itthipanichpong. Statistical analysis was performed by Napatpong Thamrongskulsiri. The first draft of the manuscript was written by Napatpong Thamrongskulsiri. All authors critically reviewed and revised the manuscript for important intellectual content and approved the final version for submission.

## CONFLICT OF INTEREST STATEMENT

The authors declare no conflicts of interest.

## ETHICS STATEMENT

This study complies with ethical standards. Not applicable.

## Supporting information

Appendix A.

## Data Availability

The data used in this study were obtained from published articles, which were cited in the references section.
