# Supplementary material for: Early mobilisation versus delayed protocols after reverse total shoulder arthroplasty for nonfracture indications: A systematic review and meta‐analysis
Source: J Exp Orthop. 2025 Oct 8;12(4):e70449. doi: 10.1002/jeo2.70449 (PMC12505189; doi:10.1002/jeo2.70449)
Supplement: Supplementary file 1 — Appendix A. [file JEO2-12-e70449-s001.docx]

**Appendix A** Search Strategies

PubMed: ("reverse total shoulder arthroplasty"[Title/Abstract] OR "reverse shoulder arthroplasty"[Title/Abstract] OR "reverse shoulder replacement"[Title/Abstract] OR "RTSA"[Title/Abstract]) AND ("early mobilization"[Title/Abstract] OR "delayed mobilization"[Title/Abstract] OR "rehabilitation"[Title/Abstract] OR "immobilization"[Title/Abstract])

Limits: inception – May 23, 2025; English language

Ovid MEDLINE: (reverse total shoulder arthroplasty OR reverse shoulder arthroplasty OR reverse shoulder replacement OR RTSA) AND (early mobilization OR delayed mobilization OR rehabilitation OR immobilization)

Limits: inception – May 23, 2025; English language

Scopus: TITLE-ABS-KEY ("reverse total shoulder arthroplasty" OR "reverse shoulder arthroplasty" OR "reverse shoulder replacement" OR RTSA) AND TITLE-ABS-KEY ("early mobilization" OR "delayed mobilization" OR rehabilitation OR immobilization)

Limits: inception – May 23, 2025; English language
